# Comparative effectiveness and safety of rituximab versus subsequent anti–tumor necrosis factor therapy in patients with rheumatoid arthritis with prior exposure to anti–tumor necrosis factor therapies in the United States Corrona registry

**DOI:** 10.1186/s13075-015-0776-1

**Published:** 2015-09-18

**Authors:** Leslie R. Harrold, George W. Reed, Robert Magner, Ashwini Shewade, Ani John, Jeffrey D. Greenberg, Joel M. Kremer

**Affiliations:** Department of Orthopedics, University of Massachusetts Medical School, 55 Lake Ave North, Worcester, MA 01532 USA; Corrona, LLC, 352 Turnpike Rd, Suite 325, Southborough, MA 01772 USA; Genentech, Inc, 1 DNA Way, South San Francisco, CA 94080 USA; New York University School of Medicine, 550 1st Ave, New York, NY 10016 USA; Albany Medical Center and The Center of Rheumatology, 1367 Washington Ave, Suite 101, Albany, NY 12206 USA

## Abstract

**Introduction:**

Patients with active rheumatoid arthritis (RA) despite anti–tumor necrosis factor(anti-TNF)agent treatment can switch to either a subsequent anti-TNF agent or a biologic with an alternative mechanism of action, such as rituximab; however, there are limited data available to help physicians decide between these 2 strategies. The objective of this analysis was to examine the effectiveness and safety of rituximab versus a subsequent anti-TNF agent in anti-TNF–experienced patients with RA using clinical practice data from the Corrona registry.

**Methods:**

Rituximab-naive patients from the Corrona registry with prior exposure to ≥1 anti-TNF agent who initiated rituximab or anti-TNF agents (2/28/2006-10/31/2012) were included. Two cohorts were analyzed: the trimmed population (excluding patients who fell outside the propensity score distribution overlap) and the stratified-matched population (stratified by 1 vs ≥2 anti-TNF agents, then matched based on propensity score). The primary effectiveness outcome was achievement of low disease activity (LDA)/remission (Clinical Disease Activity Index ≤10) at 1 year. Secondary outcomes included achievement of modified American College of Rheumatology (mACR) 20/50/70 responses and meaningful improvement (≥0.25) in modified Health Assessment Questionnaire (mHAQ) score at 1 year. New cardiovascular, infectious and cancer events were reported.

**Results:**

Estimates for LDA/remission, mACR response and mHAQ improvement were consistently better for rituximab than for anti-TNF agent users in adjusted analyses. The odds ratio for likelihood of LDA/remission in rituximab versus anti-TNF patients was 1.35 (95 % CI, 0.95-1.91) in the trimmed population and 1.54 (95 % CI, 1.01-2.35) in the stratified-matched population. Rituximab patients were significantly more likely than anti-TNF patients to achieve mACR20/50 and mHAQ improvement in the trimmed population and mACR20 and mHAQ in the stratified-matched population. The rate of new adverse events per 100 patient-years was similar between groups.

**Conclusions:**

In anti-TNF–experienced patients with RA, rituximab was associated with an increased likelihood of achieving LDA/remission, mACR response and physical function improvement, with a comparable safety profile, versus subsequent anti-TNF agent users.

**Trial registration:**

ClinicalTrials.gov NCT01402661. Registered 25 July 2011.

**Electronic supplementary material:**

The online version of this article (doi:10.1186/s13075-015-0776-1) contains supplementary material, which is available to authorized users.

## Introduction

Rheumatoid arthritis (RA) is a chronic, debilitating disease characterized by persistent synovitis and systemic inflammation. When untreated or uncontrolled, RA can cause significant pain, functional disability and decreased quality of life, and increased risk of death [1]. Nonbiologic disease-modifying antirheumatic drugs (nbDMARDs), such as methotrexate (MTX), are the mainstay of therapy and the first class of agents to be used. In patients with active RA despite nbDMARD therapy, treatment guidelines recommend either step-up to combination DMARD therapy or initiation of a biologic agent.

The first choice of biologic therapy is typically an anti-tumor necrosis factor(anti–TNF) agent [2]. While anti-TNF agents have been shown in large randomized controlled trials (RCTs) to be effective at improving the signs and symptoms of RA, and preventing damage as identified on radiography, between 30 and 40 % of patients in clinical trials and real-world practice settings develop an inadequate response to anti-TNF agents, either due to a primary lack of response or secondary treatment failure due to drug resistance or intolerance [3–6]. Patients with active disease despite anti-TNF therapy can subsequently switch to either a different anti-TNF agent or a biologic agent with an alternative mechanism of action (MOA), such as rituximab. Currently, limited data are available to physicians trying to decide between these two strategies.

Rituximab, a chimeric monoclonal antibody that depletes CD20+ B cells, in combination with MTX has demonstrated sustained efficacy and a well-characterized, long-term safety profile in patients with RA who have had an inadequate response to anti-TNF agents [7, 8]. The dose for rituximab in combination with MTX is 2 × 1000 mg administered by intravenous infusions separated by 2 weeks (one course) every 24 weeks or based on clinical evaluation, but not sooner than every 16 weeks. The addition of rituximab and other non-anti-TNF agents to the anti-RA armamentarium has increased the treatment options available to patients who have failed to respond to previous anti-TNF therapy. Although there have been no RCTs directly comparing the effectiveness of rituximab with that of a subsequent anti-TNF agent in these patients, this issue has been studied in routine clinical practice in a few observational trials from Europe [9–14]; however, comparative effectiveness data are limited for the use of rituximab in patients in the USA. As previously reported, certain clinical characteristics (e.g., disease duration, autoantibody seropositivity, comorbidities and smoking prevalence) and treatment patterns, including dosing of biologic agents and use of prednisone, can vary widely between patients in the USA compared with European registries, which may impact study results [15, 16]. In addition, access to biologic agents may differ from country to country based on payer or regulatory restrictions, further highlighting the need for USA-specific data. The objective of this analysis was to evaluate the effectiveness and safety of rituximab compared with that of a subsequent anti-TNF agent in patients with RA who had prior anti-TNF exposure, using clinical practice data from the Corrona registry.

## Methods

### Data source

The Corrona registry is an independent, prospective, observational cohort of patients with RA, who were recruited at >160 private and academic practice sites across 40 states in the USA; additional details have been published previously [17]. Data on approximately 39,950 patients with RA have been collected as of 31 March 2014. The Corrona database includes information about 285,726 patient visits and approximately 119,298 patient-years of follow up observation time, with a mean time of patient follow up of 3.6 years (median 2.8 years). For this national study, approvals for data collection and analyses were obtained from a central institutional review board (New England Institutional Review Board) for private practice sites participating within Corrona. For the <20 % of sites that are affiliated with an academic medical center, the local institutional review board was the Institutional Review Board of record.

### Study population

**Fig. 1:**
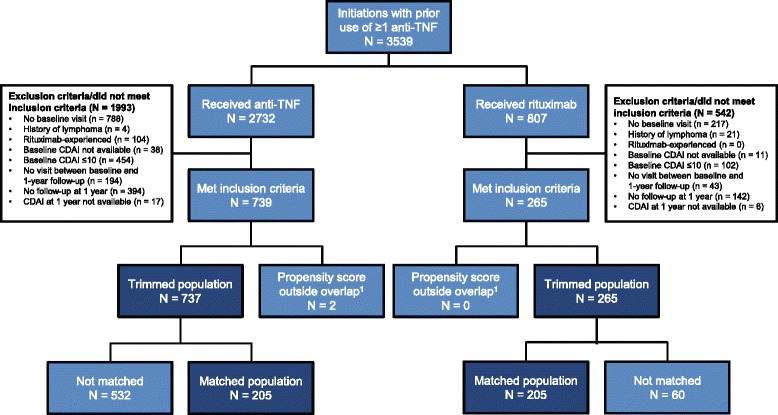
Patient disposition. ^1^Propensity scores were calculated using baseline patient demographic data, disease characteristics (severity, duration and activity), comorbidities, past medication history and concurrent medications. *Anti-TNF* anti–tumor necrosis factor agent, *CDAI* Clinical Disease Activity Index

Data were collected from patients with RA from the Corrona registry who initiated rituximab or a subsequent anti-TNF agent (adalimumab, etanercept, golimumab, infliximab, certolizumab) on or after 28 February 2006. The study population was limited to patients who had received ≥1 anti-TNF agent and had not previously received rituximab. Patients must have had the following data available to be included in the study: date of the first rituximab infusion or initiation of a subsequent anti-TNF agent; follow-up visit at 1 year (±3 months); ≥1 visit between baseline and 1-year follow up; and Clinical Disease Activity Index (CDAI) measurements at baseline and 1-year follow up. For patients whose anti-TNF initiation occurred between visits, a prior visit (within 4 months of initiation) was used. Patients with CDAI low disease activity (LDA) or remission at initiation, or with a diagnosis of lymphoma prior to initiation, were excluded from the study (Fig. 1). All patients provided written informed consent prior to participation.

### Measures and data collection

Data from Corrona were collected during the study period (28 February 2006 to 31 October 2012) from physician and patient questionnaires completed during routine clinical encounters. Data on use of nbDMARDs and biologic DMARDs, 28-joint tender and swollen joint counts, physician and patient global assessments of disease activity, patient assessment of pain and modified Health Assessment Questionnaire (mHAQ) scores assessing physical function were recorded at the time of the clinical encounter [18]. Data on demographics, insurance status, comorbid conditions, RA disease characteristics and RA medications were available for ≥99 % of patients.

### Drug exposure cohorts

To balance for predisposing factors that may increase a patient’s likelihood of receiving either rituximab or an anti-TNF agent, a propensity score - or the probability of treatment selection - was calculated for each eligible patient using baseline (at the time of drug initiation) patient demographics (age, sex, race and insurance type), disease characteristics (rheumatoid factor (RF) seropositivity, duration of RA, American Rheumatism Association functional class, tender and swollen joint counts, patient and provider global assessments, patient pain and functional status), comorbidities (cardiovascular disease, cancer and/or diabetes mellitus), past medication history (number of prior nbDMARDs, anti-TNF agents and/or non–anti-TNF agents) and concurrent medications (prednisone and/or MTX). The rationale for the methodology for this approach is provided in the supplementary materials (see Additional file 1). For the first cohort (trimmed population), patients who fell outside the overlap of the propensity score distributions were excluded (see Additional file 2: Figure S1). The second cohort (stratified-matched population) included rituximab-treated and anti-TNF-agent–treated patients who were stratified by prior treatment with one versus two or more anti-TNF agents, and then matched within each stratum based on propensity score estimated within each strata without replacement, using calipers of 0.01. The resulting stratified-matched population resulted in greater similarity between the two drug exposure groups.

### Study outcomes

The primary outcome was the proportion of patients in each group who achieved CDAI LDA or remission (CDAI score ≤10) at 1 year [18]. Secondary outcomes included the proportion of patients who achieved modified American College of Rheumatology (mACR) 20/50/70 responses, which omit the acute-phase reactant laboratory components, and the proportion who achieved a clinically meaningful improvement in functional status, defined as a decrease of ≥0.25 from baseline in the mHAQ score, at 1 year [19–21].

Safety events reported by providers over the 12-month study were examined and included infections (all infections and serious infections), cardiovascular events and new malignancies. Infections identified in this analysis included cellulitis, sinusitis, diverticulitis, sepsis, pneumonia, bronchitis, gastroenteritis, meningitis, encephalitis, urinary tract infection, upper respiratory tract infection, tuberculosis, joint infection, bursal infection and all other hospitalized and ambulatory infections. Cardiovascular events included cardiac arrest, congestive heart failure, myocardial infarction, coronary artery disease, unstable angina, ventricular arrhythmia, cardiac revascularization stroke, transient ischemic attack and deep vein thrombosis. Cancer events included breast cancer, lung cancer, lymphoma, skin cancer (not specified, squamous cell and melanoma) and other cancer diagnoses.

### Analysis and statistical methods

Patients were included regardless of retreatment with rituximab or persistence with anti-TNF therapy. Baseline patient demographics and clinical and disease characteristics were compared between the two drug-exposure cohorts, and standardized differences were estimated. Response was defined as achievement of primary and secondary outcomes at 1 year regardless of continuation of initial treatment. Nonresponse was imputed for patients who switched biologic agents. Descriptive statistics were used to examine rates of response at 1 year overall and by treatment pattern subgroup: (1) patients who remained on the drug, (2) those who were not retreated with rituximab or who discontinued anti-TNF therapy and did not initiate another biologic agent, and (3) those who switched to another biologic agent.

Multivariable logistic regression models were fit to estimate odds ratios (ORs) and 95 % CIs comparing response rates in rituximab users to anti-TNF agent users in the two populations. Covariates used in the multivariable logistic regression models of the trimmed population included baseline parameters with a standardized difference of >0.1 and four factors chosen a priori to ensure no residual confounding despite the propensity score methodology: baseline CDAI score, steroid use (current or not), number of anti-TNF agents previously used (1 vs ≥2) and concomitant MTX use. The resulting multivariable logistic regression models were adjusted for fixed and random effects. Both patient and provider random effects were examined; however, only patient-related random effects were included in the model because provider random effects did not have a significant impact on responses. In the stratified-matched population, all baseline characteristics had standardized differences <0.1 except for baseline CDAI score (standardized difference 0.14). Therefore, logistic regression models were fit to estimate ORs and 95 % CIs comparing response with rituximab to that with anti-TNF agents, including baseline CDAI score as a covariate in the model and random effect for matched pairs (i.e., patients clustered within matched pairs).

Of the patients with available data on RF status, RF seropositivity was reported in 82 % (141 of 173) and 72 % (315 of 435) of patients receiving rituximab and anti-TNF agents, respectively. Of note, a missing indicator was used when RF was included as a variable in the multivariate model as well as the propensity score model. Inclusion of RF status as a covariate resulted in <5 % variation in OR estimates. Due to the impact on power of limiting the study sample to only those for whom serologic data were available, the ORs reported do not include this covariate. Additionally, no significant differences were observed in prednisone and nbDMARD use between users of rituximab or anti-TNF agents overall, or between subgroups in the stratified-matched population. Because no significant differences were found, we chose not to include these factors in the trimmed population (because any difference would likely be controlled for in the multivariable model) and stratified-matched analyses.

Safety event rates were calculated based on the number of events reported by providers, divided by the duration of exposure. In the trimmed population, sex- and age-standardized adverse event (AE) rates among rituximab users were calculated based on the age and sex distribution in the users of anti-TNF agents. Safety events were also compared in the stratified-matched population. The ratio of rates for the rituximab users in relation to the users of anti-TNF agents was generated for both the trimmed and stratified-matched populations.

## Results

### Baseline demographics

A total of 265 rituximab users and 739 users of subsequent anti-TNF agents met the inclusion criteria prior to implementation of the propensity scores (Fig. 1). Two patients receiving anti-TNF (one with low and one with high propensity scores) were excluded from the trimmed population, leaving patients on 265 rituximab and 737 on anti-TNF agents for analysis. Approximately 16.2 % of rituximab users and 29.4 % of users of anti-TNF agents switched to another biologic agent. Among rituximab users, 21.5 % were not retreated compared with 19.1 % of patients on anti-TNF agents who discontinued their drug without initiating a new biologic agent. The stratified-matched population included 205 patients each in the rituximab and anti-TNF groups. Of the users of rituximab and anti-TNF agents, 15.1 % and 34.6 %, respectively, switched to a new biologic agent. The proportion of patients who were not retreated with rituximab was 22.9 %. The proportion of patients on anti-TNF agents who discontinued the anti-TNF treatment but did not initiate another biologic agent was 19.5 %.

Baseline characteristics of patients in the trimmed and stratified-matched populations are presented in Table 1. In the trimmed population, rituximab users had a longer duration of disease, worse disease activity scores and prior exposure to a greater number of nbDMARDs and biologic agents than users of anti-TNF agents.

**Table 1:** Baseline demographics and disease characteristics among patients receiving rituximab or anti-TNF agents, and standardized differences

	Trimmed population			Stratified-matched population		
	Rituximab (*n* = 265)	Anti-TNF (*n* = 737)	Standardized difference	Rituximab (*n* = 205)	Anti-TNF (*n* = 205)	Standardized difference
Demographics						
White, %	87.6	83.5	0.114	84.4	87.3	0.084
Female, %	81.1	79.2	0.049	82.9	81.0	0.051
Age, mean (SD), years	57.8 (11.7)	56.1 (12.4)	0.139	57.6 (11.7)	58.0 (11.5)	0.037
Insurance						
Medicare, %	37.7	29.4	0.176	33.7	39.0	0.111
Medicaid, %	7.2	6.4	0.032	7.3	6.3	0.039
Private insurance, %	76.6	78.7	0.050	76.1	74.6	0.034
No insurance, %	1.1	2.3	0.090	1.5	1.5	0.000
History of comorbidities						
Cardiovascular disease, %	11.3	7.3	0.137	9.8	11.7	0.063
Cancer, %	9.4	7.7	0.061	10.2	8.8	0.050
Diabetes, %	9.8	10.7	0.030	10.2	10.7	0.016
Clinical characteristics						
Disease duration, mean (SD), years	15.4 (10.2)	11.4 (9.4)	0.408	14.6 (10.3)	15.1 (10.5)	0.047
Tender joint count, mean (SD)	9.6 (7.3)	8.7 (7.3)	0.117	9.5 (7.2)	9.1 (7.8)	0.058
Swollen joint count, mean (SD)	8.0 (5.6)	7.5 (5.7)	0.097	7.9 (5.5)	7.4 (5.8)	0.086
Patient global assessment, mean (SD)	54.3 (23.6)	50.6 (25.0)	0.154	53.2 (23.4)	52.7 (25.5)	0.023
Physician global assessment, mean (SD)	42.7 (20.2)	39.3 (20.1)	0.169	42.3 (19.7)	39.8 (19.1)	0.130
Patient pain, mean (SD)	56.5 (24.5)	53.2 (25.5)	0.132	55.3 (24.8)	53.0 (26.2)	0.093
Disability index (mHAQ), mean (SD)	0.78 (0.5)	0.64 (0.5)	0.270	0.74 (0.5)	0.72 (0.5)	0.040
Clinical Disease Activity Index, mean (SD)	27.3 (12.5)	25.2 (11.9)	0.172	27.0 (12.0)	25.7 (12.3)	0.100
Prior medication use						
Prior nbDMARDs, n, mean	1.6	1.1	0.396	1.4	1.4	0.031
Prior use of ≥2 anti-TNF agents, %	63.0	26.6	0.786	54.6	54.6	0.000
Prior use of non–anti-TNF biologic agents, %	40.8	13.0	0.657	31.7	33.2	0.031
Concomitant medications						
Prednisone, %	43.8	29.3	0.303	39.0	38.5	0.010
Methotrexate, %	57.7	55.9	0.037	57.1	51.7	0.108
Non-methotrexate nbDMARD, %	29.1	24.8	0.095	29.8	30.2	0.011

*Anti-TNF* Anti–tumor necrosis factor, *mHAQ* modified Health Assessment Questionnaire, *nbDMARD* nonbiologic disease-modifying antirheumatic drug

### Rates of LDA or remission at 1 year

Overall rates of LDA or remission at 1 year and by subgroup (those who remained on drug, those who stopped treatment or those who switched) in both the trimmed and stratified-matched populations are presented in Table 2. In the trimmed population, 34.3 % of patients (91 of 265) receiving rituximab achieved LDA or remission at 1 year versus 33.7 % of patients (248 of 737) receiving an anti-TNF agent (*P* = 0.82). In the stratified-matched population, the unadjusted rates of LDA or remission in the rituximab and anti-TNF groups were 36.6 % (75 of 205) and 28.8 % (59 of 205), respectively (*P* = 0.09).

**Table 2:** Unadjusted response rates for LDA or remission at 1 year in all patients and by subgroup in the trimmed and stratified-matched populations

	Trimmed population				Stratified-matched population			
	Rituximab		Anti-TNF agent		Rituximab		Anti-TNF	
	(*n* = 265)		(*n* = 737)		(*n* = 205)		(*n* = 205)	
	Patients, number	LDA or remission	Patients, number	LDA or remission	Patients, number	LDA or remission	Patients, number	LDA or remission
Overall, n (%)^*^	265	91 (34.3)	737	248 (33.7)	205	75 (36.6)	205	59 (28.8)
Remained on drug	165	71 (43.0)	379	196 (51.7)	127	59 (46.5)	94	45 (47.9)
Not retreated with rituximab or discontinued anti-TNF	57	20 (35.1)	141	52 (36.9)	47	16 (34.0)	40	14 (35.0)
Switched	43	N/A	217	N/A	31	N/A	71	N/A

^*^ *P* = 0.82 for the trimmed population; *P* = 0.09 for the stratified-matched population. *Anti-TNF* anti–tumor necrosis factor, *LDA* low disease activity, *N/A* not available

**Fig. 2:**
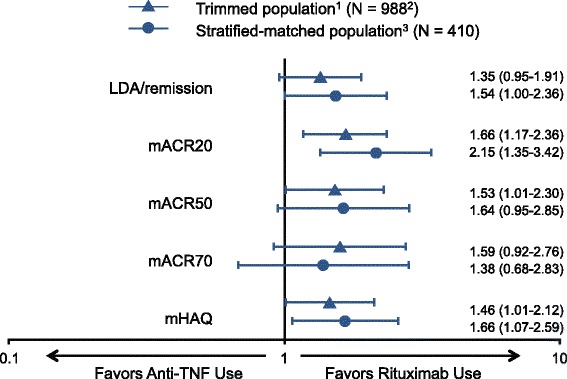
Adjusted odds ratio (OR) for effectiveness outcomes in the trimmed and stratified-matched populations. ^1^Adjusted for baseline demographics, disease activity, comorbidity and medication use (past and current). ^2^Patients with missing covariate information (n = 14) were excluded. ^3^Adjusted for disease activity. *Anti-TNF* anti–tumor necrosis factor, *LDA* low disease activity, *mACR* modified American College of Rheumatology criteria, *mHAQ* modified Health Assessment Questionnaire

In the adjusted logistic regression models, no significant differences were found in the likelihood of achieving LDA or remission between patients in the trimmed population treated with rituximab and those treated with anti-TNF agents (Fig. 2; OR, 1.35; 95 % CI, 0.95, 1.91). In the stratified-matched population, patients who received rituximab were significantly more likely to achieve LDA or remission (OR, 1.54; 95 % CI, 1.00, 2.36).

### Rates of secondary outcome measures at 1 year

Unadjusted rates of secondary outcome measures for the two treatment groups are presented in Table 3. In the trimmed population, a greater proportion of patients receiving rituximab achieved greater mACR20/50/70 responses at 1 year than those who received a subsequent anti-TNF agent (mACR20, 36.6 % vs 28.7 %; mACR50, 21.1 % vs 17.4 %; mACR70, 10.2 % vs 8.8 %), although the mACR50 and mACR70 comparisons were not significant. Similarly, a greater proportion of rituximab users achieved a clinically meaningful improvement in mHAQ score at 1 year compared with users of anti-TNF agents (33.2 % vs 24.2 %). In the stratified-matched population, rates of mACR20 (38.1 % vs 22.0 %), mACR50 (21.0 % vs 13.7 %) and mACR70 responses (10.2 % vs 7.3 %) and mHAQ score improvement (34.2 % vs 23.9 %) were generally consistent with respective rates observed in the trimmed population; crude differences between rituximab users and their stratified-matched anti-TNF counterparts were of greater magnitude.

**Table 3:** Unadjusted rates of mACR20/50/70 and mHAQ improvement

Outcome, %	Trimmed population			Stratified-matched population		
	Rituximab	Anti-TNF	*P* value	Rituximab	Anti-TNF	*P* value
	(*n* = 265)	(*n* = 737)		(*n* = 205)	(*n* = 205)	
mACR20	36.6	28.7	0.02	38.1	22.0	0.001
mACR50	21.1	17.4	0.17	21.0	13.7	0.005
mACR70	10.2	8.8	0.46	10.2	7.3	0.29
mHAQ	33.2	24.2	0.004	34.2	23.9	0.02

*Anti-TNF* anti–tumor necrosis factor, *mACR* modified American College of Rheumatology criteria, *mHAQ* modified Health Assessment Questionnaire

In a multivariate analysis of the trimmed population, patients who received rituximab were significantly more likely than those who received a subsequent anti-TNF agent to achieve mACR20 (Fig. 2; OR 1.66; 95 % CI 1.17, 2.36), mACR50 (OR 1.53; 95 % CI 1.01, 2.30) and mHAQ score improvement (OR 1.46; 95 % CI 1.01, 2.12) but were not significantly more likely to achieve mACR70 (OR 1.59; 95 % CI 0.92, 2.76). In the stratified-matched population, rituximab users were more likely to achieve mACR20 (OR 2.15; 95 % CI 1.35, 3.42) and clinically meaningful improvement in mHAQ score (OR 1.66; 95 % CI 1.07, 2.59) than those who received a subsequent anti-TNF agent (Fig. 2). No significant differences were found between the treatment groups in the likelihood of achieving mACR50 (OR 1.64; 95 % CI 0.95, 2.85) or mACR70 (OR 1.38; 95 % CI 0.68, 2.83). For both the primary and secondary outcomes, type of therapy (defined as monotherapy, combination therapy with MTX and combination therapy with a non-MTX DMARD) was not a moderating factor.

### Safety

The rates of AEs in the two populations are reported in Table 4. The standardized rates per 100 person-years for cancer, infection and cardiovascular events in patients on rituximab versus anti-TNF agents were 1.5 versus 1.9, 37.7 versus 41.0 and 1.8 versus 1.4, respectively. No significant differences were observed between the two groups. Similar rates were reported in the stratified-matched population. More detailed information on the number and type of events and available patient-years is summarized in Additional file 3: Table S1.

**Table 4:** Rates of adverse events

Events/100 PY (95 % CI)	Trimmed population^a^			Stratified-matched population		
	Rituximab	Anti-TNF	Ratio of	Rituximab	Anti-TNF	Ratio of rates^b^
	(*n* = 265)	(*n* = 737)	rates^b^	(*n* = 205)	(*n* = 205)	
Cancer	1.5 (0.0, 3.0)	1.9 (0.8, 3.0)	0.8 (0.3, 2.3)	1.6 (0.5, 4.9)	2.9 (1.2, 7.0)	0.5 (0.1, 2.8)
Infection						
All events	37.7 (29.9, 45.5)	41.0 (36.0, 46.0)	0.9 (0.7, 1.2)	38.1 (30.2, 48.0)	44.4 (35.4, 55.5)	0.9 (0.6, 1.2)
Serious infections	1.2 (0.0, 2.7)	2.8 (1.3, 4.3)	0.4 (0.1, 1.3)	2.0 (0.7, 6.3)	4.5 (2.0, 10.0)	0.5 (0.1, 2.1)
Cardiovascular	1.8 (0.2, 3.4)	1.4 (0.5, 2.4)	1.3 (0.4, 4.0)	1.6 (0.5, 4.9)	2.9 (1.2, 7.0)	0.5 (0.1, 2.8)

^a^Rates in the trimmed population were standardized by age and sex. Confidence intervals were calculated using a Poisson approximation of the standard error. ^b^Ratio of rituximab/anti-TNF agent rates. *Anti-TNF* anti–tumor necrosis factor, *PY* patient-years

## Discussion

While anti-TNF agents are the most widely used biologic drugs in RA, not all patients respond adequately, and limited data are available to rheumatologists to rationally determine subsequent therapy in anti-TNF–experienced patients. Due to the scarcity of head-to-head RCTs, several observational studies have been conducted to analyze this important clinical question. This is the first observational study in a USA population to assess the comparative effectiveness of rituximab versus that of an anti-TNF agent among anti-TNF-experienced patients with moderate-to-severe disease. In this study, patients from real-world US rheumatology practices were enrolled. To minimize selection bias, we identified the drug exposure cohorts by propensity score, excluding those who fell outside the area of common support (trimmed population) and propensity score–matched patients stratified by number of prior anti-TNF agents (stratified-matched population). In adjusted analyses of two drug-exposure cohorts categorized using propensity scores, patients treated with rituximab were 35 to 50 % more likely to achieve LDA or remission than those treated with a subsequent anti-TNF agent, although this was not statistically significant in the trimmed population. Patients treated with rituximab were also more likely to achieve mACR20 and mACR50 responses (trimmed population only) and demonstrate clinically meaningful improvement in mHAQ scores than those treated with a subsequent anti-TNF agent. We also found similar drug-related safety in the two treatment groups - comparable to published reports [15] - which is essential information for providers when deciding the next therapeutic step.

The results of this study reinforce the important observations from European studies that switching to rituximab is superior to receiving another anti-TNF agent, and expand upon these findings with a rigorous evaluation of the comparative safety of these two drug classes, consistent with the Institute of Medicine definition of comparative effectiveness [22]. This is important because patients and the rheumatologists treating them need a comprehensive evaluation of the benefit − risk profiles of different biologic agents to optimize decision-making. In a Swiss cohort study, rituximab versus an alternative anti-TNF agent was associated with a greater improvement in disease activity over 6 months of treatment, regardless of the number of previous anti-TNF agent exposures [9]. In a Dutch cohort study, patients who previously failed to respond to two anti-TNF agents demonstrated greater improvement in disease activity at 1 year after treatment with rituximab versus treatment with a third anti-TNF agent [12]. In the Spanish MIRAR study, switching to rituximab versus subsequent adalimumab or infliximab significantly improved disease activity [11]. No additional benefit was demonstrated when rituximab was compared with pooled anti-TNF agents, suggesting that the difference in response was based on the specific anti-TNF agent used [11]. In a previous Corrona study, rates of response and remission with a subsequent anti-TNF agent did not differ by mechanism of anti-TNF blockade [23]; thus, this stratification was not performed in the patients on anti-TNF therapy. Studies of European patients with RA from the British Society for Rheumatology Biologics Register and from Spanish and Dutch cohorts have also found greater improvement in physical function after switching to rituximab versus an alternative anti-TNF agent [11, 12, 14].

We believe that the findings of this study have major clinical relevance for practicing rheumatologists. Given the growing number of biologic agents indicated for RA, with their myriad mechanisms of action, comparative data are especially important for rheumatologists to guide decision-making to ensure that patients are achieving the best clinical outcomes. After head-to-head RCTs, observational studies are the best approach for evaluating comparative treatment outcomes. In particular, understanding treatment response when switching to an agent with an alternative MOA versus within a drug class is essential when selecting treatments. This study adds to the body of knowledge because it compared B-cell depletion to TNF-α pathway blockade. Prior studies demonstrating similar effectiveness between a subsequent anti-TNF agent and abatacept, a selective co-stimulation modulator, in patients with RA suggests that changing the MOA is not enough to result in improved outcomes; rather, it is the specific target of therapy that matters [24].

This study had several strengths. It is the largest known comparative effectiveness study of rituximab versus anti-TNF therapy in the USA and included a nationwide sampling of patients with RA. This is important because patient characteristics have been shown to differ between American and European registry studies, reinforcing the need for complementary USA- and Europe-based effectiveness data [15]. The all-comers study design recruited individuals from multiple rheumatology centers, resulting in a range of patients with real-world disease activity and comorbidities not often seen in RCTs. In addition to evaluating both patient- and physician-reported outcome measures, robust methodology was used. Whereas prior studies have excluded patients who switched biologic agents and censored observations after treatment interruptions, response in this study was evaluated at 1 year in all patients, regardless of continuation of initial treatment, and imputed nonresponse for switchers only [10]. This method has been described previously and was adopted to generate a more conservative estimate of effectiveness [25]. It accurately accounts for real-world situations when a drug may be considered discontinued (e.g., not retreating with rituximab), despite continuing to be beneficial to patients. Because retreatment with rituximab was at the discretion of the provider, the estimates are likely conservative. Additionally, comparative effectiveness was broadly defined, with evaluation of response, function and safety between the two drug-exposure cohorts, which had not been done in the European cohorts. All three elements are required when communicating with patients. Physicians need to discuss both the benefits (likelihood of response and improved patient-reported functional outcomes) and the risks (AEs) with patients for shared decision-making.

This study also had some limitations related to the challenges of operationalizing available, real-world data and applying analytical methods conservatively. There is always concern that patients enrolled in registries may not reflect the type of patients observed elsewhere in general practice; however, this is a general limitation of all real-world observational studies. A previous study demonstrated that de-identified data from a subset of patients in Corrona shared similar demographic and clinical characteristics with those patients in the USA Medicare population who filed rheumatology-based claims, suggesting that data from Corrona may be generalizable to the RA population in the USA [26]. The sample size included patients with prior exposure to between one and three anti-TNF agents. Because previous studies have shown that patients who have failed to respond to only one prior biologic agent respond significantly better to a new agent than those who have failed to respond to two or more biologic agents, this may have introduced bias if the number of prior anti-TNF agents differed by drug-exposure status [27]. Rituximab users were matched to patients on anti-TNF agents with the same number of prior exposures to anti-TNF agents to address this limitation. However, this did result in a smaller sample size. Also, as in any observational study, bias is a concern because physicians prescribe therapies based on the patient’s profile, and treatment selection is not random. To overcome this limitation, two cohorts were analyzed: patients with propensity scores within the area of common support and those matched by propensity score and stratified by prior use of anti-TNF therapy. Due to sample size considerations, sensitivity analyses were not performed based on reasons for discontinuation of the prior anti-TNF agent, although other studies have shown that this can influence treatment response [10]. It is possible that the occurrence of a serious AE may influence the likelihood of whether a patient receiving an anti-TNF agent switches to a biologic agent with an alternate MOA, such as rituximab, or initiates another anti-TNF agent. However, given the risks associated with rituximab treatment, it was not expected that there would be differential prescribing patterns favoring one agent vs another based on AEs associated with the initial anti-TNF therapy. Similarly, while a greater proportion of patients on anti-TNF therapy switched biologic therapies compared to rituximab users, thereby influencing the results, there was no reason to believe that physicians used different criteria for switching. Outcomes were evaluated over a 12-month period, allowing both treatment groups ample time to switch agents if they were not experiencing substantial improvement based on MOA.

## Conclusions

In conclusion, patients who switched to rituximab demonstrated a greater benefit in terms of achieving LDA or remission, mACR20/50 response and improvement in physical function, compared with patients who received a subsequent anti-TNF agent. These results are aligned with the results seen in clinical trials [28]. Finally, safety profiles for the two treatment groups were similar and consistent with what has been previously reported [7]. Taken together, these results suggest that in clinical practice, rituximab may be more efficacious than a subsequent anti-TNF agent in patients with moderately active to severely active RA and prior exposure to anti-TNF agents. Future analyses are necessary to better identify which patients are likely to respond to a particular agent based on their response to prior medications.

## Additional files

Additional file 1:
**Supplementary methods.** Covariates included in the final models in the stratified-matched population, and explanation of the propensity score methodology and rationale for use of this approach. (DOCX 34 kb) Additional file 2: Figure S1. Propensity score distributions for anti-TNF and rituximab use in patients in the trimmed population who previously received one anti-TNF agent (a) and those who previously received two or more anti-TNF agents (b). (PPTX 78 kb) Additional file 3: Table S1. Rates of adverse events (trimmed population) in patients receiving rituximab or anti-TNF agents. (DOCX 24 kb)

## Abbreviations

AE: adverse event
anti–TNF: anti–tumor necrosis factor
CDAI: Clinical Disease Activity Index
CI: confidence interval
LDA: low disease activity
mACR criteria: modified American College of Rheumatology criteria
mHAQ: modified Health Assessment Questionnaire
MOA: mechanism of action
MTX: methotrexate
nbDMARD: nonbiologic disease-modifying antirheumatic drug
OR: odds ratio
RA: rheumatoid arthritis
RCT: randomized controlled trial
RF: rheumatoid factor
