# Supplementary material for: Comparative effectiveness and safety of rituximab versus subsequent anti–tumor necrosis factor therapy in patients with rheumatoid arthritis with prior exposure to anti–tumor necrosis factor therapies in the United States Corrona registry
Source: Arthritis Res Ther. 2015 Sep 18;17(1):256. doi: 10.1186/s13075-015-0776-1 (PMC4574482; doi:10.1186/s13075-015-0776-1)
Supplement: Additional file 1: — Supplementary methods. Covariates included in the final models in the stratified-matched population, and explanation of the propensity score methodology and rationale for use of this approach. (DOCX 34 kb) [file 13075_2015_776_MOESM1_ESM.docx]

**ADDITIONAL FILE 1**

**Analysis and statistical methods**

The covariates included in the final models in the stratified-matched population were age, white race, duration of rheumatoid arthritis, insurance type (Medicare, Medicaid, private insurance and no insurance), American Rheumatism Association functional class, baseline Clinical Disease Activity Index, patient pain, modified Health Assessment Questionnaire, history of cardiovascular disease, past medication history (≥2 prior anti–tumor necrosis factor agents [anti-TNFs], prior non–anti-TNF use and number of prior nonbiologic disease-modifying antirheumatic drugs [nbDMARDs]), concurrent medications (prednisone and methotrexate) and rheumatoid factor (RF) seropositivity (ever).

**Explanation of propensity score methodology**

Overview of propensity scores

Propensity score statistical techniques are increasingly being used to address questions of causal inference in rheumatology. The choices of propensity models, potential methods using the resulting scores, and comparison to regression techniques are outlined by Austin (2011) and summarized here [S1]. Rosenbaum and Rubin (1983) defines the propensity score as “*the conditional probability of assignment to a particular treatment given a vector of observed covariates* [S2]*.*” Several analytic choices are made as part of this process, and no definitive consensus on a “best” method for all analyses exists. Austin (2011) provides some guidance based on review of the literature [S1].

Variable selection for the propensity model

Austin (2011) indicates a lack of consensus but concludes that “…*in many settings, it is likely that one can safely include all measured baseline characteristics in the propensity score mode* [S1]*.*” Brookhart et al (2006) state that variables unrelated to exposure but related to outcome should be included for precision, while variables related to exposure but unrelated to outcome may decrease the precision of the exposure effect [S3]. Furthermore, in small studies, variables strongly related to exposure but weakly related to outcome might be detrimental. Austin (2011) in turn indicates the reality that “*it may be difficult to accurately classify baseline variables into the true confounders…*[S1]*.*”

Balance diagnostics

There is consensus that sample size–dependent tests (e.g., *t* tests) are not appropriate diagnostics and that standardized differences should be determined using the equation [S4]:

where *s* is the pooled variance. For dichotomous outcomes, this is based on the estimate proportions:

It is less clear when balance of propensity scores is close enough between 2 exposure groups. Austin (2011) points out that Normand (2001) in a study used 0.1 to represent a negligible difference, basing it on Cohen’s interpretation of effect size (Cohen 1988) [S1, S5, S6].

Propensity score methods

Several methods have been proposed for using the propensity scores to adjust for confounding:

- *Matching*: Matched sets of the 2 treatments are created so that the patients share similar values of the propensity score. Austin (2011) explains that one-to-one matching is the most common approach. Matching can be done with or without replacement [S1]. “Without replacement” matches unique patients—once a patient has been matched, he or she is no longer available for matching to another patient. Matching “with replacement” creates the potential for the same patient to be matched to multiple patients, but variance estimates must account for this in the analysis. Either greedy matching or optimal matching can be done. In greedy matching, a patient in 1 exposure group is chosen at random and matched to a patient in the other exposure group whose propensity score is the closest. Optimal matching creates matches that minimize the total within-pair differences. Gu and Rosenbaum (1993) found that optimal matching “*…is no better than greedy matching in the sense of producing balanced matched samples* [S7]*.*” Matching can be carried out with no limit on “closeness” in propensity scores between matched pairs or by using a caliper with the maximum difference in propensity scores that is allowed. Austin (2011) indicates that “*…there is no uniformly agreed upon definition of what constitutes a maximal acceptable difference* [S1]*.*” It usually presents a trade-off in the number of matches and the balance. Rubin and Thomas (2000) show that propensity matching can be combined with additional regression adjustment to account for residual confounding [S8].
- *Stratification*: Patients are stratified into mutually exclusive subsets based on ranges of the propensity score. A common method is to create quintiles (or deciles, depending on sample size). Within each stratum, the treatment effect on outcome is estimated and then pooled across the stratum. As with the matched analysis, within-stratum regression adjustment can be used.
- *Inverse probability weighting*: This method uses the propensity scores to weight the populations, similar to survey sampling weights. Balance is determined by examining the weighted baseline characteristics. Variance estimates must account for the weighting (as in survey weights), and instability may arise from very low propensity scores. Methods for stabilizing the weights have been proposed by Robins et al (2000) [S9].
- *Covariate adjustment using propensity score*: Regression adjustment using the propensity score is estimated. This combines the regression of the outcome with the propensity score directly. It is basically a data reduction technique to produce a single covariate for the regression.
- *Regression adjustment after propensity trimming*: This method accomplishes the usual adjustment of the treatment and outcome association using regression models, including confounding covariates. The propensity score trimming ensures that the patients in each treatment group have “common support.” The regression adjustment will not extrapolate to patients in either group who have characteristics that are not in common with those in the other treatment group. If the excluded (trimmed) population is large, it should be compared to the included populations in order to understand the restricted population to which the results apply.

Austin (2011) provides a summary of comparison of the various methods and indicates that “*Several studies have demonstrated propensity score matching eliminates a greater proportion of the systematic differences in baseline characteristics…than does stratification on the propensity score or covariate adjustment using the propensity score,*” suggesting that matching and inverse probability weighting “*removed systematic differences…to a comparable degree* [S1].”

Rationale for the propensity score methods used in this manuscript

The proposed methods for this paper include the use of 2 different methods to examine the robustness of results under different models. The first is the propensity trimming regression model, which is closest to the standard regression model but excludes patients at extremes that would include extrapolation of results to dissimilar patients. The second is a propensity matching method that uses one-to-one and greedy matching with a caliper. In this study, the “stratified-matched population” was composed of rituximab and anti-TNF users who were stratified by 1 versus ≥2 prior anti-TNFs and then matched within each stratum based on propensity score estimated within each stratum, without replacement, using a caliper of 0.01. The resulting stratified-matched population resulted in greater similarity between the 2 drug-exposure groups. This is one of the propensity methods suggested by Austin that will provide elimination of a greater proportion of systematic differences in baseline characteristics.

**SUPPLEMENTAL REFERENCES**

S1. Austin PC. An introduction to propensity score methods for reducing the effects of confounding in observational studies. *Multivariate Behav Res* 2011;46:399–424.

S2. Rosenbaum PR, Rubin DR. The central role of the propensity score in observational studies for causal effects. *Biometrika* 1983;70:41–55.

S3. Brookhart MA, Schneeweiss S, Rothman KJ, *et al*. Variable selection for propensity score models. *Am J Epidemiol* 2006;163:1149–56.

S4. Flury BK, Riedwyl H. Standard distance in univariate and multivariate analysis. *Am Stat* 1986;40:249–51.

S5. Normand ST, Landrum MB, Guadagnoli E, *et al*. Validating recommendations for coronary angiography following acute myocardial infarction in the elderly: a matched analysis using propensity scores. *J Clin Epidemiol* 2001;54:387–98.

S6. Cohen J. *Statistical Power Analysis for the Behavioral Sciences*. Hillsdale, NJ: Lawrence Erlbaum Associates; 1988.

S7. Gu XS, Rosenbaum PR. Comparison of multivariate matching methods: structures, distances and algorithms. *J Comp Graph Stat* 1993;2:405–20.

S8. Rubin DB, Thomas N. Combining propensity score matching with additional adjustments for prognostic covariates. *JASA* 2000;95:573–85.

S9. Robins JM, Hernan MA, Brumback B. Marginal structural models and causal inference in epidemiology. *Epidemiology* 2000;11:550–60.
