# Supplementary material for: Comparative effectiveness and safety of rituximab versus subsequent anti–tumor necrosis factor therapy in patients with rheumatoid arthritis with prior exposure to anti–tumor necrosis factor therapies in the United States Corrona registry
Source: Arthritis Res Ther. 2015 Sep 18;17(1):256. doi: 10.1186/s13075-015-0776-1 (PMC4574482; doi:10.1186/s13075-015-0776-1)
Supplement: Additional file 2: Figure S1. — Propensity score distributions for anti-TNF and rituximab use in patients in the trimmed population who previously received one anti-TNF agent (a) and those who previously received two or more anti-TNF agents (b). (PPTX 78 kb) [file 13075_2015_776_MOESM2_ESM.pptx]

## Slide 1
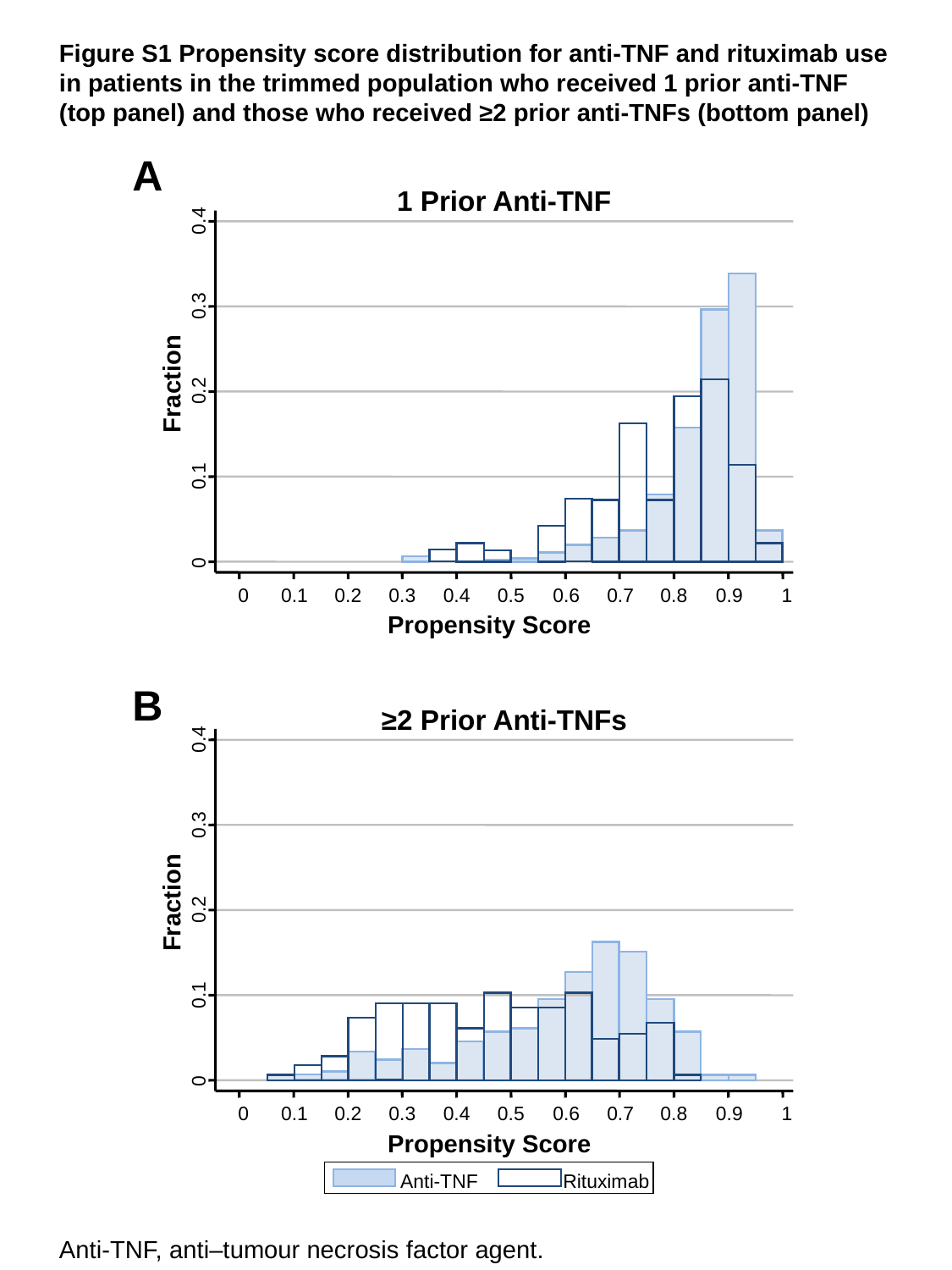

Figure S1 Propensity score distribution for anti-TNF and rituximab use in patients in the trimmed population who received 1 prior anti-TNF (top panel) and those who received ≥2 prior anti-TNFs (bottom panel)
A
1 Prior Anti-TNF
0.4
0.3
Fraction
0.2
0.1
0
0
0.1
0.2
0.3
0.4
0.5
0.6
0.7
0.8
0.9
1
Propensity Score
B
≥2 Prior Anti-TNFs
0.4
0.3
Fraction
0.2
0.1
0
0
0.1
0.2
0.3
0.4
0.5
0.6
0.7
0.8
0.9
1
Propensity Score
Anti-TNF
Rituximab
Anti-TNF, anti–tumour necrosis factor agent.
