# Supplementary material for: Comparative effectiveness and safety of rituximab versus subsequent anti–tumor necrosis factor therapy in patients with rheumatoid arthritis with prior exposure to anti–tumor necrosis factor therapies in the United States Corrona registry
Source: Arthritis Res Ther. 2015 Sep 18;17(1):256. doi: 10.1186/s13075-015-0776-1 (PMC4574482; doi:10.1186/s13075-015-0776-1)
Supplement: Additional file 3: Table S1. — Rates of adverse events (trimmed population) in patients receiving rituximab or anti-TNF agents. (DOCX 24 kb) [file 13075_2015_776_MOESM3_ESM.docx]

**ADDITIONAL FILE 3**

**Table S1 Rates of adverse events (trimmed population)**

|  | **Rituximab Patients**  **(n = 265)** | | | **Anti-TNF Patients**  **(n = 737)** | | |
| --- | --- | --- | --- | --- | --- | --- |
| **Adverse event** | **Events** | **PY** | **Events/100 PY**  **(95% CI)** | **Events** | **PY** | **Events/100 PY**  **(95% CI)** |
| **Cancer** |  |  |  |  |  |  |
| **All events** | 4 | 242.4 | 1.6 (0.6-4.4) | 12 | 634.1 | 1.9 (1.1-3.3) |
| **Skin** | 3 | 242.4 | 1.2 (0.4-3.8) | 7 | 634.1 | 1.1 (0.5-2.3) |
| **Lung** | 1 | 184.2 | 0.4 (0.1-2.9) | 0 | 494.5 | 0.0 (0.0-0.7)^1^ |
| **Breast** | 0 | 184.2 | 0.0 (0.0-2.0)^1^ | 1 | 494.5 | 0.2 (0.0-1.1) |
| **Lymphoma** | 0 | 242.4 | 0.0 (0.0-1.5)^1^ | 0 | 634.1 | 0.0 (0.0-0.6)^1^ |
| **Other** | 0 | 242.4 | 0.0 (0.0-1.5)^1^ | 4 | 634.1 | 0.6 (0.2-1.7) |
| **Infection** |  |  |  |  |  |  |
| **All events** | 93 | 242.4 | 38.4 (31.3-47.0) | 260 | 634.1 | 41.0 (36.3-46.3) |
| **Serious infections** | 3 | 184.2 | 1.6 (0.5-5.0) | 14 | 494.5 | 2.8 (1.7-4.8) |
| **Cardiovascular** |  |  |  |  |  |  |
| **All events** | 5 | 242.4 | 2.1 (0.9-5.0) | 9 | 634.1 | 1.4 (0.7-2.7) |

^1^One-sided, 97.5% CI.

Anti-TNF, anti–tumor necrosis factor agent; PY, patient-year(s).
